# Exploring associations between multipollutant day types and asthma morbidity: epidemiologic applications of self-organizing map ambient air quality classifications

**DOI:** 10.1186/s12940-015-0041-8

**Published:** 2015-06-23

**Authors:** John L. Pearce, Lance A. Waller, James A. Mulholland, Stefanie E. Sarnat, Matthew J. Strickland, Howard H. Chang, Paige E. Tolbert

**Affiliations:** Department of Public Health Sciences, College of Medicine, Medical University of South Carolina, 135 Cannon Street, Charleston, SC 29422 United States; Department of Biostatistics and Bioinformatics, Rollins School of Public Health, Emory University, Atlanta, GA United States; Department of Environmental Health, Rollins School of Public Health, Emory University, Atlanta, GA United States; School of Civil and Environmental Engineering, Georgia Institute of Technology, Atlanta, GA United States

## Abstract

**Background:**

Recent interest in the health effects of air pollution focuses on identifying combinations of multiple pollutants that may be associated with adverse health risks.

**Objective:**

Present a methodology allowing health investigators to explore associations between categories of ambient air quality days (i.e., multipollutant day types) and adverse health.

**Methods:**

First, we applied a self-organizing map (SOM) to daily air quality data for 10 pollutants collected between January 1999 and December 2008 at a central monitoring location in Atlanta, Georgia to define a collection of multipollutant day types. Next, we conducted an epidemiologic analysis using our categories as a multipollutant metric of ambient air quality and daily counts of emergency department (ED) visits for asthma or wheeze among children aged 5 to 17 as the health endpoint. We estimated rate ratios (RR) for the association of multipollutant day types and pediatric asthma ED visits using a Poisson generalized linear model controlling for long-term, seasonal, and weekday trends and weather.

**Results:**

Using a low pollution day type as the reference level, we found significant associations of increased asthma morbidity in three of nine categories suggesting adverse effects when combinations of primary (CO, NO_2_, NO_X_, EC, and OC) and/or secondary (O_3_, NH_4_, SO_4_) pollutants exhibited elevated concentrations (typically, occurring on dry days with low wind speed). On days with only NO_3_ elevated (which tended to be relatively cool) and on days when only SO_2_ was elevated (which likely reflected plume touchdowns from coal combustion point sources), estimated associations were modestly positive but confidence intervals included the null.

**Conclusions:**

We found that ED visits for pediatric asthma in Atlanta were more strongly associated with certain day types defined by multipollutant characteristics than days with low pollution levels; however, findings did not suggest that any specific combinations were more harmful than others. Relative to other health endpoints, asthma exacerbation may be driven more by total ambient pollutant exposure than by composition.

**Electronic supplementary material:**

The online version of this article (doi:10.1186/s12940-015-0041-8) contains supplementary material, which is available to authorized users.

## Introduction

Currently, there is much scientific interest in investigations of multiple pollutants in air pollution health studies to fill a general lack of knowledge surrounding the impacts of multiple pollutants and health [1–5]. It is anticipated that quantification of such ‘multipollutant’ health risks will more accurately reflect the etiologic relationships between air pollution and adverse health and that certain combinations of pollutants may be found to be more toxic than others for particular outcomes [2]. It is important to note that this knowledge gap is not the result of lack of understanding of how air pollution exposure occurs (i.e., via inhalation of complex pollutant mixtures) but rather the result of limitations of traditional epidemiologic models and exposure characterization methodologies [6, 7]. Factors such as the strong multicollinearity between different pollutants present in most air pollution data sets present inferential challenges since standard statistical analyses will typically result in inflation of standard errors. In response, several promising methodologies for characterizing multiple pollutants and examining multipollutant health risks appear in the environmental epidemiology literature [8–12]; however, a recent review by Oakes, Baxter et al. (2014) notes that there is no gold standard for multipollutant exposure characterization or health effects estimation and that much remains to be learned [7].

In order to fill this knowledge gap it is clear that more research on the development and application of multipollutant exposure metrics in health studies is needed. For example, it is still largely unknown whether or not multipollutant metrics provide a reasonable explanation of air pollution health effects or if they provide any improvement upon single pollutant metrics. The reliability of many multipollutant methods, as well as potential impacts of exposure characterization error and confounding, remain uncertain.

To address this problem, we focus on multipollutant features driving local air quality (in this case at the city level). Different weather elements (such as temperature, humidity, wind speed, and boundary layer height) and pollution sources interact in locally characteristic and distinct manners with local air quality. Therefore, understanding of such features on a local scale could play an important role in the development of a multipollutant exposure characterization. For example, if a study found that the daily occurrence of a particular multipollutant combination has stronger impacts on health than others, we might conclude that further studies of this combination are needed. However, if this event only occurs on a small fraction of days in the study (e.g., < 1 %), investigators may be somewhat less concerned about development of more complex methods needed for further investigation.

In the present study, we explore short-term associations between multiple pollutants and emergency department (ED) visits for pediatric asthma by addressing the following research questions:

- What multipollutant combinations are present on days in our study period and how often do these multipollutant day types occur? What do ‘typical’ multipollutant day types look like and what do ‘rare’ or ‘extreme’ multipollutant day types look like?
- How does each multipollutant day type observed in our study associate with health? Do certain day types associate more strongly with adverse health than others?

Our overarching goal in addressing these questions is to increase our understanding of how multipollutant combinations associate with acute pediatric asthma morbidity and to provide insight into development of methods that can be useful for guiding future efforts aimed at exploring risks associated with exposure to multiple pollutants.

## Methods

To address our first set of research questions, we apply an unsupervised learning tool known as the self-organizing map (SOM) [Pearce, Waller et al. (2014) explain the use of SOMs for ambient air quality classification] to develop categories of days (i.e., multipollutant day types) based on ambient air quality data that reflect how multipollutant combinations vary in time at our study location. To address our second set of research questions we apply a time-series epidemiologic model [13] to estimate associations of multipollutant day types and pediatric asthma morbidity. We also perform a sensitivity analysis exploring the impact of our choice of number of categories and compare results to single pollutant models.

### Developing multipollutant day types

The SOM algorithm [14] applies an unsupervised learning process to project features discovered in input data onto the elements of a regular one- or two-dimensional array (i.e., the ‘map’) in an organized fashion. In addition to providing an efficient means of interpreting and visualizing complex multipollutant data sets, the ‘map’ facilitates understanding of between-class relationships – a characteristic not available with traditional techniques [10]. The way SOM is used here is similar to cluster analysis [8]; however, we tailor our approach towards identifying a collection of multipollutant day types that can be used as independent exposure variables rather than the discovery of ‘distinct’ profiles in our data.

We applied SOM to daily measures of ten air pollutants collected at a central air monitoring location in an industrial and commercial area in downtown Atlanta in order to identify categories of days that reflect the temporal variation in multipollutant conditions observed from January 1, 1999 to December 31, 2008. Pollutants included in the analysis included measures for 1-hr maximum carbon monoxide (CO) in ppm, 1-hr maximum nitrogen dioxide (NO_2_) and nitrous oxides (NO*x*) in ppb, 8-hr maximum ozone (O_3_) in ppb, 1-hr maximum sulfur dioxide (SO_2_) in ppb, and five 24-hr average PM_2.5_ components in μg/m^3^: elemental carbon (EC), organic carbon (OC), nitrate (NO_3_), ammonium (NH_4_), and sulfate (SO_4_). For more detail on application of SOM see Additional file 1.

### Selecting the number of multipollutant day types

With SOM, as well as for other clustering techniques, the choice of the number of categories is an important step that must be determined by the user [10]. Although several procedures exist for identifying groups in data [15], the issue has yet to be formalized for our purposes and thus we guide our decision using criteria we believe support an exploratory epidemiologic investigation. As such, our aims are to identify a number of day types that minimizes the information lost in our exposure of interest yet retains enough power and precision for statistical inference. To achieve this, we begin by applying principal components analysis (PCA) to our data to identify the primary modes of variance as well as provide a means to graphically explore grouping structure. Next, we evaluate the distribution of variance explained in our data as a function of class number as information loss is a concern. Lastly, we evaluate the relative frequency distribution of multipollutant day type assignments as a function of category number to better understand sample size considerations. Results are used collectively to assist in selecting a number of multipollutant day types for use in our epidemiologic analysis. It is important to note that the unsupervised nature of our study is exploratory and thus our intention is to identify day types that can inform hypothesis generation for further research not ‘ideal’ risk categories [16].

### Health outcome data

We obtained aggregate daily counts for pediatric asthma related emergency department (ED) visits for children ages 5 to 18 years from 41 hospitals within metropolitan Atlanta for our study period. We defined ED visits for pediatric asthma as all visits with a code for asthma (493.0–493.9) or wheeze (786.07) using the International Classification of Diseases, 9^th^ Revision.

### Epidemiologic modeling

We modeled associations between multipollutant day types and pediatric asthma emergency department visits using a case-crossover design within the framework of a Poisson generalized linear model allowing for overdispersion. The general framework of our model is similar to those applied in previous studies of Atlanta data [9, 11, 13]. In brief, the dependent variable was the daily number of pediatric asthma emergency department visits and the primary exposure variable was a categorical variable with indicators for each multipollutant day type. To control for potential confounding, the model included indicator variables for year, season, month, day-of-the-week, hospital, and holidays, their interactions; and, to control for weather, cubic polynomial terms for three-day averages of mean temperature and mean dew point temperature. Models also included interactions between temperature and season.

The estimated main effects are rate ratios (RRs) comparing the adjusted pediatric asthma rate for each multipollutant day type as compared with that under the referent day type. In order to maximize the contrasts between day types in our analysis the referent group was specified as the SOM category with the lowest overall pollutant concentrations. This comparison was chosen to approximate the effect of a given day type versus a relatively ‘clean air’ referent category.

As a sensitivity analysis we compare the magnitude and stability of estimated associations as a function of class number by running our epidemiologic model using output from classifications with 2 categories to a maximum of 20 categories. Finally, to discuss our findings in context with traditional approaches we present single pollutant RRs for interquartile range increases in each pollutant.

## Results

### Selecting the number of multipollutant day types

**Fig. 1:**
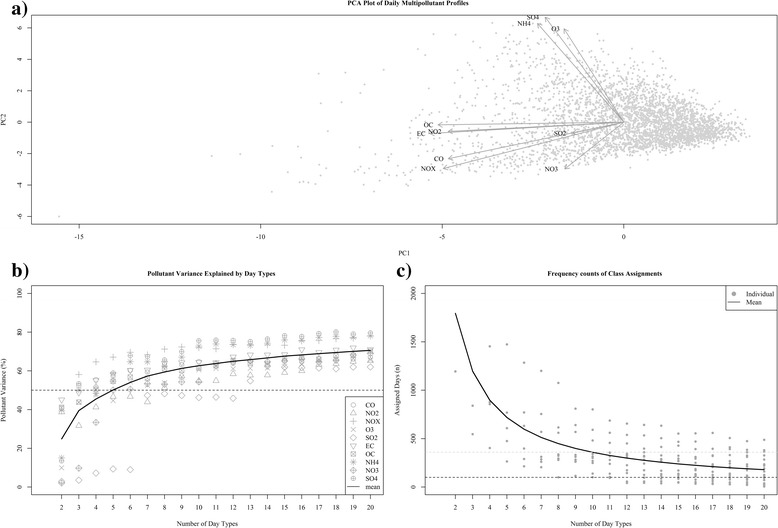
Graphical and statistical evaluation measures used to aid in selection of number of day types. Panel **a** presents a principal components analysis (PCA) projection of our multipollutant data. The *grey points* represent the scores for daily observations along the first two principal components and the *dark arrows* indicate the corresponding loading vectors for each pollutant. Panel **b** displays the distribution of adjusted R^2^ values from simple regression models fit to each pollutant as a function of the number of day types. Each pollutant has a unique symbol and the *trend line* reflects the mean. Panel **c** displays the distribution of frequency assignments to each day type. *Grey points* reflect observed frequencies and *trend line* reflects the mean

Plotting a PCA projection of our data (Fig. 1a) in combination with the component loading weights reveals a primary mode of variation (PC1) dominated by CO, NO_2_, NO_*X*_, EC, and OC, a subset of pollutants indicative of primary pollution, and a primary mode of variation (PC2) weighted towards SO_4_, NH_4_, and O_3_, marking it as a measure of secondary pollution. The relative lengths and direction of the SO_2_ and NO_3_ loading weights suggest behaviors independent from these two primary modes of variance (which capture approximately 65 % of the variance in our data). Although strong grouping is not evident in this display, PCA suggests that at least 4 separate modes of variance are needed to capture the primary features in this dataset.

Examination of the individual pollutant variance explained as a function of class number reveals a positive nonlinear relationship between class number and pollutant variation (Fig. 1b). We also see that certain pollutants (e.g., NO_X_, SO_4_, NH_4_) are better represented by the classifications than others (e.g., SO_2_, NO_3_, NO_2_), a feature that may have important implications for subsequent analysis. For our purposes, minimization of such intra-classification spread is desired in addition to a general expectation that the metric capture as much of the information available in the original data as possible. As such, we see approximately 8 groups are needed to retain half the information of the original data for 9/10 pollutants (SO_2_ being the exception) and that over 12 groups are needed to capture more than half the variance of all pollutants.

The frequency counts of class assignments shows a strong relationship between number of categories and the distribution of day types available for testing (Fig. 1c). Here a nonlinear decreasing trend illustrates how smaller numbers of categories provide larger sample sizes and more categories provides smaller sample sizes. We require a classification that retains numbers of days in each day type to be useful in further analyses and thus, to assist, we have added two reference lines to the plot – a black dashed line for a suggested minimum sample size of 100 days (which equates to ~3 % of all days) and a grey dashed line for identification of relatively rare events at a frequency of 10 % (approximately 365 individual days) during our study period. These indicators suggest we could reasonably examine all classifications containing around 10 categories or less.

Collectively, these findings improve our understanding of the variance structure of our data and reveal how different partitions of our data can be used to capture certain properties of interest for our exposure characterization (Fig. 1). Based on these findings, we determined that a classification consisting of 9 categories was sufficient to describe ambient air quality days because it has the benefit of providing a modest approximation of the original data (all R^2^ > 0.5 except SO_2_) and reasonable sample sizes (all day types > 100 days).

### Multipollutant day types

**Fig. 2:**
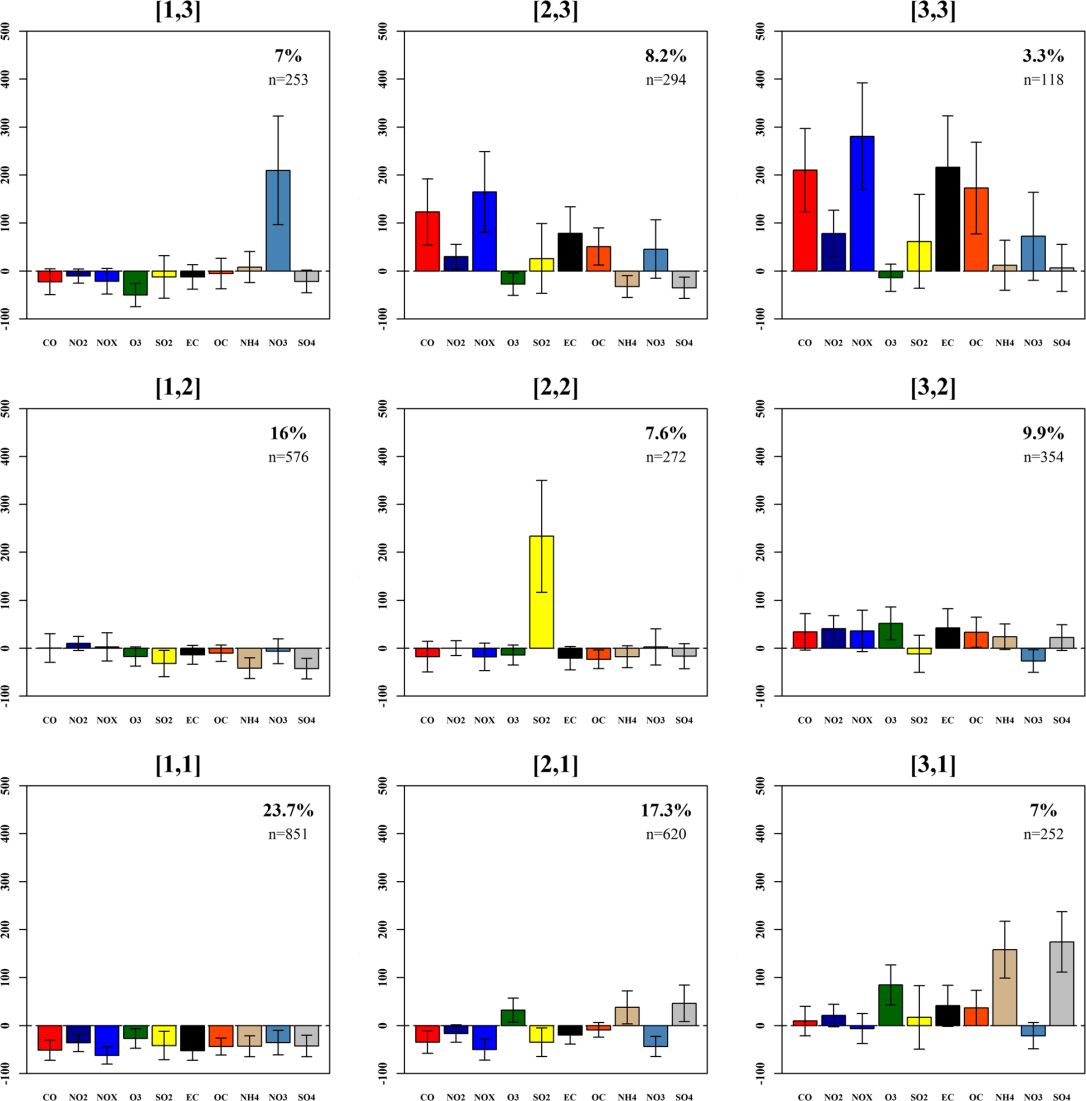
A 3 × 3 SOM of 9 multipollutant day types observed in Atlanta, GA, from January 1, 1999 to December 31, 2008. For each type, profile bars reflect the average (±SD) pollutant concentrations on assigned days zeroed to the overall mean on a percentage scale. Coordinate labels are in brackets [] and the relative frequencies (%) and within-class sample size (n) are presented in the upper right hand corner of each panel

To answer our first set of proposed questions we present a 3x3 SOM that illustrates ambient air quality using 9 categories of days that reflect the range of multipollutant events frequently observed at our monitoring location (Fig. 2). Each category is described as a multipollutant day type (MDT) and is referenced using SOM [x,y] coordinates. Furthermore, MDT profiles are displayed as barplots that present mean centered values on a percentage scale along with their standard deviations. For summary statistics of the SOM see Table 1.

**Table 1:** Means (standard deviations) for pollutant concentrations and meteorological variables in each SOM category, Atlanta, GA, 1999 to 2008

SOM [X,Y]	CO (ppm)	NO2 (ppb)	NOx (ppb)	O3 (ppb)	SO2 (ppb)	PM2.5 EC (μg/m3)	PM2.5 OC (μg/m3)	PM2.5 NH4 (μg/m3)	PM2.5 NO3 (μg/m3)	PM2.5 SO4 (μg/m3)	PM10 (μg/m3)	PM2.5 (μg/m3)	TEMP (C)	DEWTMP (C)
[1, 1]	0.5 (0.3)	26.3 (7.8)	44 (23)	31.1 (11.9)	8.5 (8.5)	0.7 (0.3)	2.3 (0.8)	0.9 (0.4)	0.6 (0.4)	2.6 (1.1)	16 (6.5)	9.1 (2.9)	16.1 (7.6)	10.5 (9.1)
[1, 2]	1.1 (0.5)	45.1 (8.7)	119.7 (55.9)	35.1 (12.9)	10 (7.7)	1.2 (0.5)	3.6 (1.1)	0.9 (0.4)	0.9 (0.5)	2.6 (1.1)	20.3 (7.6)	11.9 (3.3)	14.4 (6.3)	6.6 (8.4)
[1, 3]	0.8 (0.5)	36.7 (9.4)	91.8 (54.8)	21.2 (10.8)	12.8 (12)	1.3 (0.6)	3.8 (1.8)	1.7 (0.7)	2.8 (1)	3.6 (1.7)	20 (8)	16.1 (6.1)	6.9 (5.3)	1.2 (7.8)
[2, 1]	0.7 (0.4)	34.1 (10.2)	58.3 (31.3)	56.2 (13.5)	9.5 (9)	1.2 (0.4)	3.7 (1)	2.2 (0.6)	0.5 (0.3)	6.7 (1.9)	28.2 (7.1)	18.7 (4.2)	24.2 (3.7)	17.1 (4.2)
[2, 2]	0.9 (0.6)	41 (10.5)	95.4 (57)	36.5 (16.1)	48.8 (17.1)	1.1 (0.6)	3.1 (1.1)	1.3 (0.6)	0.9 (0.6)	3.8 (2.1)	20.6 (8.1)	13.2 (5.3)	13.8 (8.9)	5.8 (10.5)
[2, 3]	2.4 (0.7)	53.3 (12.2)	308.8 (100.5)	30.9 (14.2)	18.4 (14)	2.6 (0.9)	6.1 (1.7)	1.1 (0.5)	1.3 (0.7)	3 (1.5)	27 (8.4)	18.6 (5.1)	11.9 (5.3)	3.4 (6.9)
[3, 1]	1.2 (0.5)	49.5 (13.4)	109.3 (59.2)	78.6 (18.5)	17.1 (14.3)	2 (0.7)	5.5 (1.7)	4.1 (1)	0.7 (0.4)	12.5 (2.9)	43.5 (9.9)	31.6 (5.8)	26.2 (3)	18.5 (2.9)
[3, 2]	1.4 (0.5)	57.7 (11.6)	158.8 (64.9)	64.7 (15.5)	12.9 (9.9)	2 (0.7)	5.4 (1.5)	2 (0.6)	0.7 (0.3)	5.6 (1.8)	33 (8.6)	20.6 (4.6)	21.7 (4.3)	12.6 (5.3)
[3, 3]	3.3 (0.9)	72.9 (20)	444.2 (130.2)	36.6 (19.7)	23.7 (16.3)	4.5 (1.5)	11 (3.9)	1.8 (1.2)	1.6 (0.9)	4.9 (3.2)	43.9 (16.9)	29.9 (9.6)	13.9 (5.5)	4.8 (7.4)
Overall	1.1 (0.8)	41 (15.8)	116.4 (109.1)	42.6 (21.3)	14.6 (15.1)	1.4 (1)	4 (2.3)	1.6 (1.1)	0.9 (0.8)	4.6 (3.2)	24.8 (11.7)	16.2 (7.9)	17.2 (8.1)	10 (9.1)

**Fig. 3:**
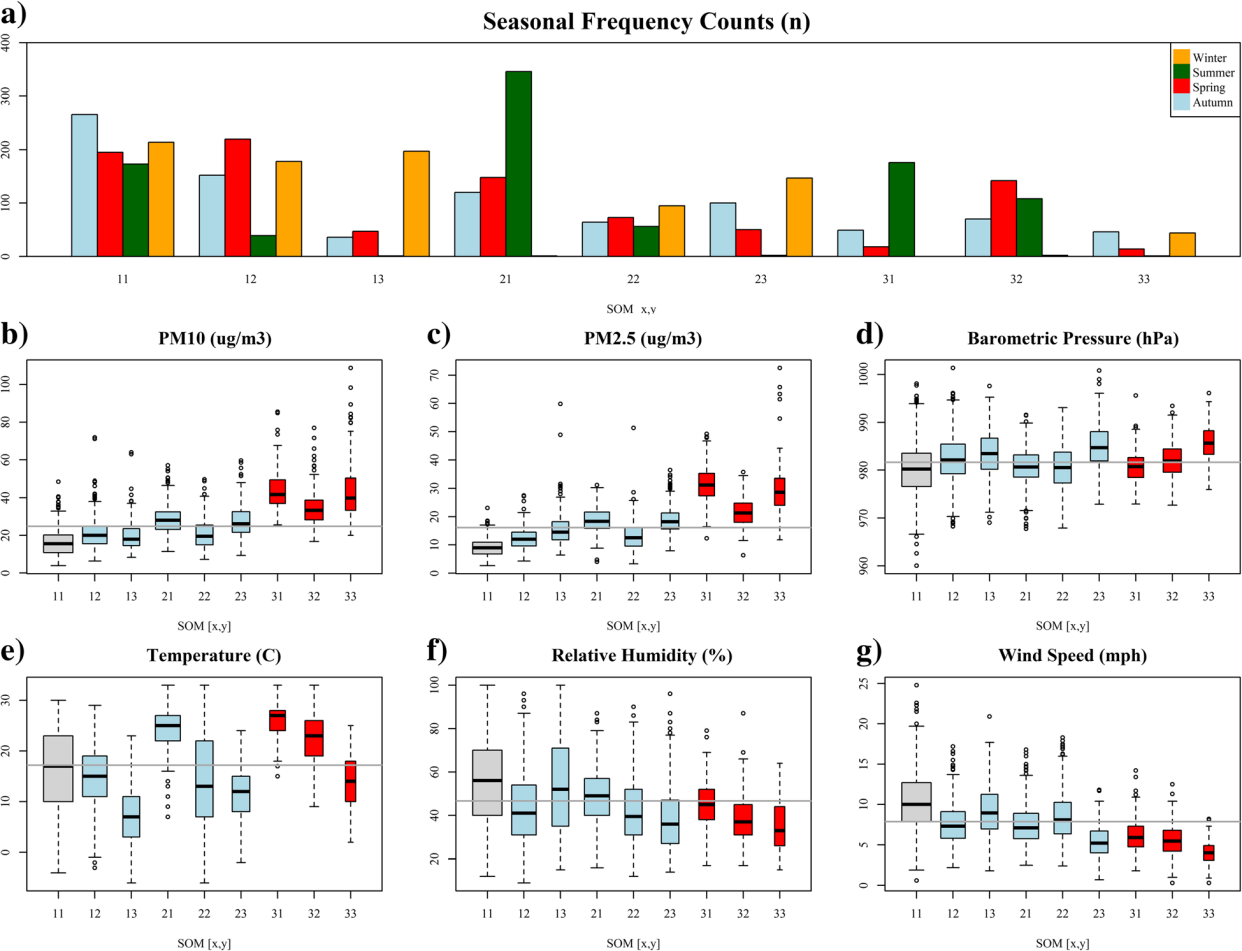
Seasonal frequencies, particulate matter, and meteorological summaries for the 9 multipollutant day types identified in Atlanta, GA, from January 1, 1999 to December 31, 2008. Panel **a** provides the seasonal frequency counts for each type. Panels **b**-**g** provide boxplots illustrating the distribution of particulate matter and meteorology under each category. *Grey* indicates our referent level; *Light blue* indicates insignificant day types; *Red* indicates day types significantly associated with asthma

We found that the most common types of days at our study location are characterized by profiles in the bottom left corner of the map (Fig. 2). The most frequent type of day (24 % of all days) in our study, MDT [1, 1], reveals that air quality conditions for Atlanta are most often well below average (i.e., the overall mean pollutant levels for Atlanta). Such relatively ‘clean’ days were found to be distributed across all seasons and were accompanied by low air pressures, higher humidity, and higher wind speed suggestive of strong atmospheric mixing and rain (Fig. 3). Another common type of day (MDT [2, 1]; 17 %) groups springtime events that entail slightly above average concentrations for secondary pollutants (O_3_, NH_4_, and SO_4_) and a relatively warm and stable atmosphere. MDT [1, 2] assembles cooler dry days with near to slightly above average concentrations for primary pollutants such as NO_2_ and NO_*X*_. Collectively, these profiles capture 57 % of days in Atlanta and thus we would consider such types to represent ‘typical’ air quality events at our study location.

Moving to other sections of the map we find types of days that were somewhat less common and indicative of extreme conditions for a variety of combinations of pollutants (Fig. 2). In the center MDT [2, 2] exemplifies 8 % of days when SO_2_ concentrations are well above average. In the upper left MDT [1, 3] captures extreme NO_3_ days that occurred 7 % of the time under cool, wet, stable conditions. This agrees well with understanding of how low temperatures and high relative humidity contribute to the formation of nitrate rich aerosols in Atlanta [17]. Moving right, we see that 11 % of days (MDT [2, 3] and [3, 3]) were dominated by well-above average to extreme conditions for several primary pollutants such as CO, NO_2_, NO_X_, EC, and OC. Consistent with these relatively cold dirty days are the high pressure, low wind speeds, and low humidity conditions suggestive of poor atmospheric mixing and potential inversions (Fig. 3). Furthermore, MDT [2, 3] and MDT [3, 3] exhibit high ratios of OC/EC (2.39 and 2.43, respectively) and thus are indicative of days dominated by mobile source emissions from gasoline [17]; however, MDT [3, 3] is far less frequent (3.3 % of days) and highlights days when several pollutants are two to three times higher than average – a scenario we might *a priori* describe as the most hazardous air quality scenario in our study.

Moving towards the right center, MDT [3, 2] represents a relatively polluted type of day when concentrations were above average for several primary and secondary pollutants. Accompanying weather was warm, dry, and stable, conditions known to promote outdoor physical activity [18]. In the bottom right, MDT [3, 1] represents days dominated by extreme concentrations for O_3_, NH_4_, and SO_4_. This chemical profile occurred primarily in summer under relatively hot, humid conditions and suggests high photochemical activity.

### Associations with pediatric asthma emergency department visits

**Fig. 4:**
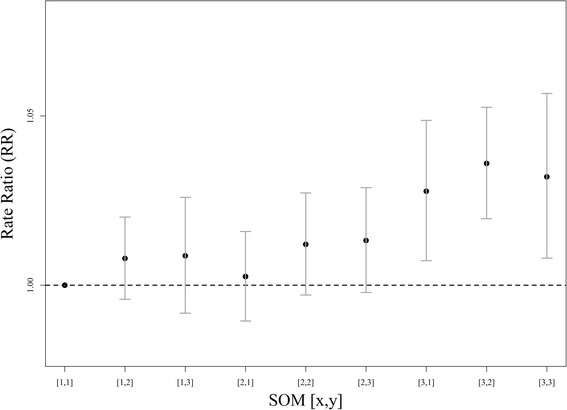
Rate ratios of emergency department visits for pediatric asthma on day following occurrence (i.e., lag 1) of each SOM-based multipollutant day type as compared to the referent group [1, 1] in Atlanta, Georgia from 1999 to 2008

The questions addressed in this section are: How does the occurrence of each type of multipollutant day observed in our study associate with the health outcome of interest? Do certain combinations associate more strongly with adverse health than others? Using MDT [1, 1] as a referent, results show significant associations with three types of days in Atlanta (Fig. 4). The strongest association (RR: 1.04, 95 % CI: 1.02, 1.05) was found for MDT [3, 2] – a type of day characterized as being generally well mixed and having above average concentrations (Fig. 2). Significant associations were also identified for MDT [3, 3] and MDT [3, 1], day types that reflect extreme concentrations for either a collection of primary pollutants (CO, NO_2_, NO_X_, EC, and OC) or a set of secondary pollutants (O3, NH_4_, and SO_4_). Collectively, these events encompassed approximately 20 % of days in our study and occurred on days when particulate matter was above average (Fig. 3). Results for MDT’s describing more typical (i.e. high frequency) air quality days ([1, 2], [2, 1]) and air quality days with single pollutant extremes ([1, 3], [2, 2]) were positive but not significant. Moderate-to-high concentration days dominated by primary pollution (MDT [2, 3]) yielded a positive estimated association but with confidence interval that includes the null; the result is intermediate between the low pollution days ([1, 2] and [2, 1]) and the high primary pollution days ([3, 3]).

### Sensitivity analysis

**Fig. 5:**
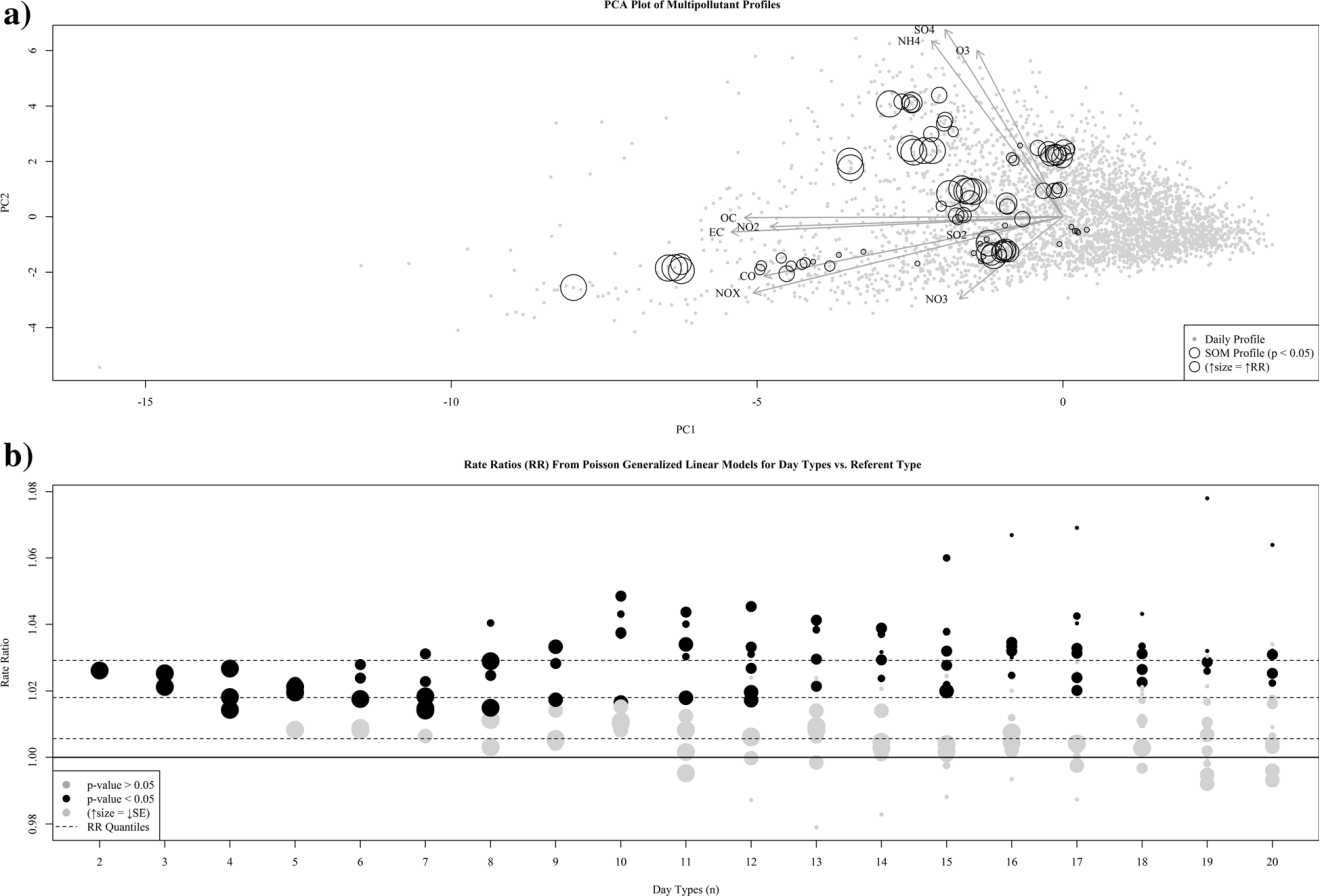
Results from sensitivity analysis of number of categories. Panel **a** presents a Principal Component Analysis biplot of the multipollutant data in our study. Vectors depict the primary modes of variation in the data (i.e., loading weights) and circles reflect estimated Rate Ratios (RR) using Poisson regression for SOM generated multipollutant profiles from a range of classifications (categories *n* = 2:20) found to be significantly associated with our outcome (*p* < 0.05). Panel **b** presents the distribution of rate ratio estimates for each SOM classification. Categories found significant (*p* < 0.05) are colored *black*, and to indicate estimate stability, size of the symbol is inversely proportional to the estimated standard error (SE). *Dashed lines* reflect quartiles for the distribution of RRs across all classifications

Results from our epidemiologic analysis with multiple SOMs revealed that RRs for multipollutant day types were somewhat sensitive to variations of class number (Fig. 5); however, based on the properties of the classifications a certain degree of variability was expected. By overlaying the SOM-derived chemical profiles found to be significantly associated with pediatric asthma from all classifications derived using a range of categories from 2 to 20 we are able to see if identification of similar types of days resulting in associations with adverse health was captured by multiple categorizations of our data (Fig. 5a). We see a general clustering of profiles significantly associated with asthma morbidity, a feature that suggests that profiles capturing similar features in the air quality data resulted in similar associations with asthma morbidity.

A strip plot of RRs for each class number reveals how the magnitude and stability of estimates vary as a function of the number of categories. Generally, we found that adding additional levels to our classifications produced categories with larger rate ratios and standard errors. These trends were somewhat expected, as the increases in magnitude of the estimates likely reflect increasing magnitude of the contrasts between tested categories and increasing standard errors correspond with decreasing sample sizes. It is important to note that we varied the referent level between the different SOMs in order to explore different ranges of contrasts and not to facilitate comparison to specific results in our 3x3 SOM. In addition to general trends, we also found that above a certain number of categories larger distinctions between the RRs became more apparent as well as emergence of ‘protective’ types of days.

### Single pollutant results

**Fig. 6:**
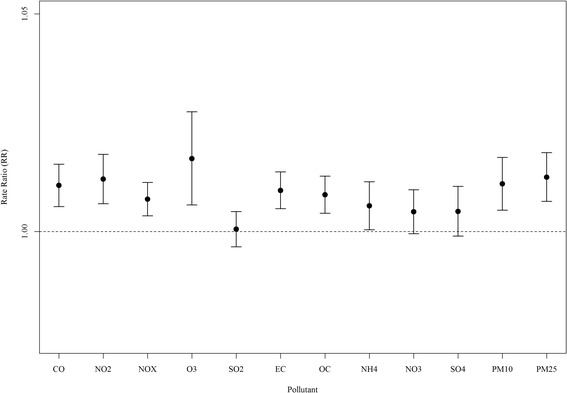
Rate ratios (RR) and 95 % confidence intervals for interquartile range increases in lag 1 ambient air pollution concentrations for single pollutants in Atlanta, Georgia from 1999 to 2008

Using single pollutant models we identified significant associations for interquartile range (IQR) increases in CO, NO_2_, NO_X_, O_3_, EC, OC, NH_4_, and PM_10_ and PM_2.5_ (Fig. 6). Overall, RR estimates for these pollutants were generally similar and agreed well with our SOM-based findings that ED visits for pediatric asthma are rather non-specific – a finding seen in previous studies [13].

## Discussion

In this study, we identified nine multipollutant day types that reflect temporal patterns in multipollutant days at our monitoring site and applied them as categories of exposure in an epidemiologic analysis. Observed daily conditions ranged from windy, wet types of days with low levels for all pollutants to generally dry, stable (i.e., low wind speeds) days with elevated pollution for either primary- or secondary-driven combinations or both types of pollutants (Figs. 2 & 3). Epidemiologic analysis identified clear associations between higher levels of pollution and adverse health. We found significant associations of increased asthma morbidity for three MDTs (Fig. 4): 1) days that were most warm, with low winds and humidity (MDT [3, 1]), and very high levels for secondary pollutants (O_3_ and ammonium sulfates); 2) days that were the driest and most stable (MDT [3, 3]), with very high primary pollutants from vehicles (CO, NOx, EC, and OC), and 3) days that were generally warm, stable and dry (MDT [3, 2]) with elevated levels for both primary and secondary pollutants. Each of these three ‘harmful’ day types was unhealthy to a similar degree. On the other extreme, wet, windy days with low pollution levels were not found to be significantly associated with asthma incidence.

Between these extremes, intermediate, non-significant associations were observed for several SOM types: warm, low-wind days when primary pollutants were elevated but not as high as MDT [3, 3] (MDT [2, 3]), cool days when nitrate was high (MDT [1, 3]), and days when SO_2_ was high (MDT [2, 2]) that likely occurred when coal combustion plumes touched down over downtown Atlanta. MDTs [1, 2] and [2, 1] were most similar to our referent group (MDT [1, 1]) both in terms of relatively low pollution levels and meteorology.

These findings indicate a greater burden of asthma morbidity on days when either primary or secondary pollutants are at their highest concentrations, and on days when both primary and secondary pollutants are moderately elevated (warm sunny days exhibiting high photochemical activity in combination with traffic emissions). Moreover, days with isolated extremes for SO_2_ or NO_3_ were modestly suggestive of adverse impacts on asthma exacerbation. Results for typical conditions indicated that normal-to-low levels of air pollution were generally not harmful. Thus, overall increases in pollution appear to adversely impact pediatric asthma exacerbation more than any unique combination, a conclusion reached by other analyses of the Atlanta data assessing this particular outcome [9, 11]. Comparison of epidemiologic results of our SOM-based exposure metric with those of single pollutant measures (Fig. 6) found general agreement between the results; however, the broader interpretation provided by the SOM findings can be informative for future studies of air pollution mixtures. In summary, we found our approach provides novel, yet simplified, results that can be used to better understand day-level multipollutant combinations and their associations with adverse health.

A key feature of this work was our demonstration of how an unsupervised learning tool (SOM) can be used to identify category boundaries in multipollutant data sets for epidemiologic investigation. We chose to deconstruct our data in such a manner because we wanted to examine contrasts in our data that emerged from patterns founded on observed environmental conditions. By centering our exposure characterization on such patterns we were able to examine contrasts in the data that emphasized distinctions in day-level concentrations amongst several pollutants not distinctions in sources or variation in health outcomes. We chose this strategy for our exposure characterization because we wanted the opportunity to investigate both the typical modes of behavior expected in air pollution (e.g., primary, secondary) and features of the system that vary in an irregular manner (e.g., SO_2,_ NO_3_, etc.). To achieve this, we tailored our approach by selecting a somewhat generous number of categories (most epidemiologic studies use categories ranging from about 2 to 5 (Royston, Altman et al. 2006)) as we were more concerned with missing a potentially important feature in our data than statistical power. In doing so, we provided our investigation the opportunity to look at more than just the global features present in the data; the generalized nature of more traditional unsupervised approaches has been noted as a limitation elsewhere [2].

The primary advantage of categorizing our multivariate data is simplicity. In using categories we were able to reduce complex relationships observed within our data into a set of representative profiles that greatly simplified our statistical analysis and produced intuitive findings that are relatively straight-forward and can be easily understood by policy makers and non-specialists in the field. Such findings give us a meaningful look at multipollutant air quality and facilitate complex health investigations by allowing joint estimation of risk for several pollutants.

Such simplicity does come at a cost (as with all multivariate reductions), however, as multivariate reduction can be seen as a form of data smoothing that results in both information and power being lost in the procedure [19]. Consequently, our findings are likely conservative and any ‘true’ associations are likely stronger than those we have reported. Nevertheless, as an exploratory tool (the intended purpose of unsupervised learning) such analyses can provide a benchmark to compare findings from more complex approaches and provide opportunity to raise new questions regarding the resulting value of multipollutant categorization for epidemiologic research. Although few, other studies have noted successes for using categorization in this setting [9, 12, 20], and our current approach adds to this literature.

As with any study design, there are opportunities for improvement. To begin with, we only considered a single lag in our investigation, and although asthma morbidity has generally noted a short-term response to air pollution [13], it is likely that we may have missed important features at other lags in our data. This raises a complex issue for multipollutant research because it is unclear how lag structures for several pollutants can be accounted for in the development of a multipollutant exposure metric. Distributed lag models have shown promise for single pollutants; however, methods aimed at identifying sequences of events could also be informative in this setting. Another potential limitation for this work is possible confounding of our results due to clear associations between several of our multipollutant day types, time of year, and local weather conditions. These findings highlight that it may be more difficult to separate confounders from multipollutant metrics than single pollutant metrics as the linkages between weather and the identified features in the air quality process are likely to be stronger than single pollutant associations. Here, we aggressively controlled for confounding through the structure of our Poisson generalized linear model [13]; however, we suggest that confounder-based categorizations are a possibility that should be explored. Another concern for our work is that we did not incorporate the uncertainty associated with our categories into our health study. Certain categorical profiles may be closer to ‘true’ conditions than others and thus it is possible that this error was propagated into our estimated health associations. Any classification procedure will return information on class quality and thus adaptations need to be made to our approach to account for this uncertainty. Source apportionment may offer opportunity for examples (e.g., positive matrix factorization) as well as approaches designed to account for exposure measurement error [21, 22]. Another limitation is that we treated each pollutant equally in this analysis and thus if associations were stronger for certain pollutants our analysis may have suppressed such relationships. We note that the nonspecificity of pollutants impacting asthma morbidity suggests this is not a major issue in our study; however, variable weighting or selection strategies could offer opportunity for improvement [23]. Finally, we only examined a single health outcome and thus other outcomes should be examined; while asthma exacerbation is robustly associated with ambient air pollution, this particular health outcome may be primarily driven by total pollutant load and, as such, may be less sensitive to pollutant composition than other health endpoints.

The natural extension of an unsupervised study is to use what has been learned in the exploratory phase to aid in the development a supervised approach that would allow development of categories more strongly associated with the outcome of interest. For example, one might suggest that ideal multipollutant categories in this setting would reflect cutpoints in the data that captured homogeneous risk within categories and heterogeneous risk between categories. With such an objective, one could formulate category boundaries that maximized resulting risk estimates or minimized P-values. However, we caution that fully supervised approaches may not be ideal given the potential for bias. For example, driving category formation with risk estimates alone would inevitably result in bias away from the null and boundaries drawn with P-values may result in a downwardly biased P-value [16]. Given such potential implications, and findings from other researchers [23–25], we anticipate that that this will continue to be an exciting area of future research.

The ever-present nature of ambient air pollution means that we are all exposed. As such, even small increases in risk (such as the ones estimated in this study) suggest that large numbers of people are impacted. It is clear that more research focused on improving our definitions of harmful exposures is needed to improve our understanding of air pollution health effects, and subsequently, improve strategies to protect public health.

## Conclusion

There is abundant interest in health effects of air pollution mixtures and additional research on this topic can facilitate improved understanding of a wide range of air pollution topics from optimal air quality management to the etiology of disease. Our analysis supports that classification can be used to develop exposure metrics that support future studies of ambient air pollution mixtures and population health.

## Additional file

Additional file 1:
**Appendix.**

## Abbreviations

SOM: Self-organizing map
PCA: Principal Component Analysis
MDT: Multipollutant Day Type
RR: Rate Ratio
