# Supplementary material for: Exploring associations between multipollutant day types and asthma morbidity: epidemiologic applications of self-organizing map ambient air quality classifications
Source: Environ Health. 2015 Jun 23;14:55. doi: 10.1186/s12940-015-0041-8 (PMC4477305; doi:10.1186/s12940-015-0041-8)
Supplement: Additional file 1: — Appendix. [file 12940_2015_41_MOESM1_ESM.docx]

**Appendix**

**SOM Algorithm**

Applying SOM requires two components – the input data matrix and the output map (Figure 1). Here, the input matrix is our multi-pollutant data set, $Z$:

$Z =\left[ \begin{matrix} z_{11} & \cdots& z_{1p} \\ \vdots& \ddots& \vdots\\ z_{n1} & \cdots& z_{np} \end{matrix} \right]$ *Eq. 1*

where $n$ denotes the number of sampling days and $p$ the number of pollutants. Each day is represented by a row $Z_{i}$ within $Z$. The output collection of class profiles is the “map”, $M$:

$M=\left[ \begin{matrix} m_{1y} & \cdots& m_{XY} \\ \vdots& ⋰ & \vdots\\ m_{11} & \cdots& m_{x1} \end{matrix} \right]$ *Eq. 2*

with each profile $m$ represented as a node at location (*x*, *y*) on the map (Figure 1). Note *X×Y* determines the number of nodes (i.e., number of classes) and the arrangement (e.g., 2D) of $M$. Topology of $M$ can be specified as either rectangular or hexagonal. Each node $m$ is associated with a vector $w_{m}:$

$w_{m}=[\mu_{m1}, \mu_{m2}, \ldots,\mu_{mp}]$ *Eq. 3*

where $\mu$ are ‘learned’ coefficient values corresponding to the pollutant concentration values that define profile $m$.

Operationally, SOM implements the following steps. First, given $M$, map initialization occurs with each $m$ being assigned a preliminary $w_{m}$ from a random selection of $Z_{i}$ ’s. Then, iterative learning begins where, for each iteration $t$, the algorithm randomly chooses a day’s profile $Z_{i}^{(t)}$ from $Z$ and then computes a measure of (dis)similarity (in our case the Euclidean distance) between the observation $Z_{i}^{(t)}$ and each $w_{m}^{(t)}$. Next, SOM provisionally assigns a best matching node $m^{*}(t)$ whose $w_{m^{*}}$ is most similar to each $Z_{i}^{(t)}$. Next, class profile development occurs via the *Kohonen learning process*:

$w_{m}^{(t+1)}=w_{m}^{(t)}+\alpha\left( t \right)N_{m^{*}i}\left( t \right)[\bar{Z}^{\left( t \right)}-w_{m}^{\left( t \right)}]$ *Eq. 4*

where $\alpha$ is the learning rate, $N_{m^{*}i}$is a neighborhood function that spatially constrains the neighborhood of $m^{*}$ on $M$, and $\bar{Z}$ is the mean of pollutant values on days provisionally assigned to the nodes within the neighborhood set. The learning rate controls the magnitude of updating that occurs for *t*. The neighborhood function, which activates all nodes up to a certain distance on $M$ from $m^{*}$, forces similarity between neighboring nodes on $M$. Equation (4) updates coefficients within a neighborhood of $m^{*}$, where the impact of the neighborhood decreases over iterations.

SOM performance is dependent on both $\alpha$ and $N$ and thus mappings are sensitive to these parameters^30^. Therefore, in effort to provide guidance we note that $\alpha$ typically starts as small number and is specified to decrease monotonically (e.g., 0.05 to 0.01) as iterations increase. Similarly, the range of $N$ starts large (e.g., 2/3 map size) and decreases to 1.0 over a predetermined termination period (e.g., 1/3 of iterations), after which fine adjustment of the map occurs.

Training continues for the number of user-defined iterations. Kohonen recommends the number of steps be at least 500 times the number of nodes on the map. Once training is complete, results include final coefficient values for each node’s $w_{m}$, classification assignments for each day $Z_{i}$, and coordinates of nodes on $M$. The final step is to visualize the class profiles by plotting the map. For additional details regarding SOM, please refer to the book of Kohonen (2001).

*SOM Implementation*

Implementation of the SOM algorithm in this study was performed using the ‘kohonen’ package in the R environment for statistical computing. For each map size, training of the SOM was accomplished by setting the algorithm to run a number of iterations equal to *n* classes × 500 for each size. The learning rate $\alpha$ and the neighborhood function *N* were kept at the default for the software – which specified $\alpha$ to decrease linearly from 0.05 to 0.01 and set *N* to start with a value that covered 2/3 of all node-to-node distances, decrease linearly, and terminate after 1/3 of the iterations had passed. A total of 10 random initializations were tested for each solution and a random initialization scheme yielding the most consistent (i.e., mode) mean square error was used for evaluation. Although several distance metrics are available, we use Euclidean distance as the (dis)similarity metric because it is considered appropriate for quantitative data. For more detail on implementation of SOM in R please refer to Wehrens and Buydens (2007).
